# The Response by International Emergency Medical Teams Following the Beirut Harbor Explosion in 2020 – Who Were They, When Did They Arrive, What Did They Do, and Were They Needed?

**DOI:** 10.1017/S1049023X22000784

**Published:** 2022-08

**Authors:** Emeli Wolff, Iman Shankiti, Flavio Salio, Johan von Schreeb

**Affiliations:** 1. Centre for Research on Health Care in Disasters, Department of Global Public Health, Karolinska Institutet, Stockholm, Sweden; 2. World Health Organization Country Office, Beirut, Lebanon; 3. Department of Country Readiness Strengthening, World Health Organization, Switzerland

**Keywords:** Beirut blast, disaster response, EMT: Emergency Medical Team, I-EMT: International Emergency Medical Team, international field hospitals, SOD: sudden-onset disaster, WHO: World Health Organization

## Abstract

**Introduction:**

On August 4, 2020, a massive explosion struck the Beirut Harbor in Lebanon. Approximately 220 people were killed and around 7,000 were injured, of which 12% were hospitalized. Despite being weakened by economic crisis and increasing numbers of coronavirus disease 2019 (COVID-19) cases, the national health care system responded promptly. Within a day, international health care assistance in the form of International Emergency Medical Teams (I-EMTs) started arriving. Previous studies have found that I-EMTs have arrived late and have not been adapted to the context and dominating health care needs. The aim of this study was to document the organization, type, activity, and timing of I-EMTs deployed to Beirut and to discuss their relevance in relation to medical needs.

**Methods:**

Data on all deployed I-EMTs were retrieved from all available sources, including internet searches, I-EMT contacts, and from the World Health Organization (WHO) EMT coordination cell (EMT CC) in Lebanon. The WHO EMT classification was used to categorize deployed teams. Information on characteristics, timing, and activities was retrieved and systematically assessed.

**Results:**

Nine I-EMTs were deployed to Beirut following the explosion. Five were equivalent to EMT Type 2 (field hospitals), out of which three were military. The first EMT Type 2 arrived within 24 hours, while the last EMT set up one month after the explosion. Four civilian I-EMTs provided non-clinical support as EMT Specialized Care Teams. A majority of the I-EMTs were focused on trauma care. Three of the four I-EMT Specialized Care Teams were rapidly re-tasked to support COVID-19 care in public hospitals.

**Conclusion:**

A majority of the deployed I-EMT Type 2 were military and focused on trauma care rather than the normal burden of disease including COVID-19. Re-tasking of EMTs requires flexible EMTs. To be better adapted, the I-EMT response should be guided by a systematic assessment of both health care capacities in the affected country as well as the varying health effects of hazards before deployment.

## Introduction

On August 4, 2020, a storage containing 2,750 tons of ammonium nitrate exploded in the Port of Beirut, Lebanon. The blast killed around 220 people, injured more than 7,000 people, and approximately 300,000 people became homeless.^1–3^ Several health facilities were destroyed^1–3^ and an estimated 500 hospital beds out of 13,000 in the country were lost.^3,4^ The Lebanese health system was already weakened by the 2019 economic collapse and increasing numbers of coronavirus disease 2019 (COVID-19) patients.^1,3^ However, the national response was prompt, impressive, and largely sufficient. Reportedly within the first two days, 8,643 injured sought care at 42 hospitals located within 88.2km of the Port of Beirut.^5^ In addition, international health care assistance started arriving within days of the explosion.

Previous studies have shown that international health care assistance after disasters has arrived late and has been ill-adapted to medical needs. International agencies have not been sufficiently coordinated, accountability has been questioned, and lack of data has made it impossible to fully evaluate their performance.^6–14^ To address these shortcomings, the World Health Organization (WHO; Geneva, Switzerland) and partners launched the Emergency Medical Team (EMT) initiative in 2013.^15^ Following guidelines on classification and standards, agencies can apply to be WHO EMT classified. The EMT initiative also includes an EMT coordination cell (EMT CC) that aims to support the Ministry of Health in the affected country to ensure that international EMTs are an asset for the national emergency operation center.

To improve international health care response to disasters, studies on recent deployments are needed. To date, the international health care assistance in Beirut has not been documented nor assessed. The aim of this study was to document the organization, type, activity, and timing of international EMTs (I-EMTs) deployed to Beirut following the explosion on August 4, 2020 and to discuss their relevance in relation to medical needs.

## Materials and Methods

This observational study compiles data collected from September 1, 2020 through February 3, 2021. No individual patient medical or personal data were included and most of the information collected was from open online sources or reports from the I-EMT Type 2s to the WHO EMT CC. Based on the EMT initiative ambition of transparency and accountability, retrieved information was treated as open source. No ethical concerns were identified that outweighed the study aim and consequently ethical review board permission was not deemed necessary.

Data were obtained based on the methodology of previous studies on I-EMT deployments.^6,7,9,11,16^ Any type of document or study written in English, related to the I-EMT response in Beirut 2020, and containing information on I-EMT type, activities, and deployment timing was included.

The data sources in this study were:

1. Secondary data made available by the WHO EMT CC in Beirut;
2. Internet searches (Google [Google Inc.; Mountain View, California USA] and PubMed [National Center for Biotechnology Information, National Institutes of Health; Bethesda, Maryland USA]) using the search words “emergency medical team,” “foreign medical team,” “field hospital,” “explosion Beirut,” “Beirut blast,” “military field hospital,” “lessons learned,” “healthcare”;
3. Key websites of deployed I-EMTs, the United Nations Office for the Coordination of Humanitarian Affairs (OCHA; New York USA) Beirut Port Explosions Situation Reports, the WHO Beirut Blast Situation Reports, and the United Nations Population Fund (UNFPA; New York USA) Situation Reports on Beirut Port Explosion;
4. Secondary data provided by a team from the Beirut Arab University (Beirut, Lebanon) on the available foreign medical capabilities in Beirut (Report on Lebanon Emergency Field Hospitals, in cooperation with UNFPA); and
5. Direct contact with the I-EMTs requesting data on their deployment.

Each I-EMT was classified as being either a governmental (civilian or military) or nongovernmental organization (NGO). The country of origin was noted. Type was defined using the EMT classification system,^17^ regardless if the agency was WHO EMT classified or not:

1. An EMT Type 1 (EMT-1) is a facility providing out-patient care,
2. An EMT Type 2 (EMT-2) offers in-patient and surgical care, and
3. An EMT Type 3 (EMT-3) offers advanced referral in-patient care.
4. Specialized Care Teams offer specialized care (such as orthoplastics or provide specific technical support) but do not offer a facility structure.^15^

Reported capacities (number and type of staff, hospital beds, and numbers of out-patient/in-patient/surgical/medical capacities) were noted as well as the date of arrival, days required for set-up, and time of departure from Beirut. Data were entered into a Microsoft Excel database (Microsoft Corp.; Redmond, Washington USA).

To ensure maximum consistency, data were triangulated and when inconsistent, they were validated using information from I-EMT staff and the WHO EMT CC. One of the authors (Johan von Schreeb) served as a WHO EMT coordinator in Lebanon and visited all I-EMTs. He held weekly I-EMT coordination meetings where the I-EMTs (except for the field hospitals, I-EMT-2) participated. His observations and official meeting reports were part of the material for the study. The WHO EMT CC also repeatedly contacted all international field hospitals (I-EMT-2) for data on activities.

## Results

### Type/Organization of I-EMTs

Nine I-EMTs were deployed to Beirut, of which five were field hospitals (EMT-2s) focused on surgical trauma care. Of these five, three were military (from Italy, Jordan, and Morocco); one governmental (Ministry of Emergency Situations of the Russian Federation – EMERCOM; Moscow, Russia); and one was from the Iranian Red Crescent (RC; Tehran, Iran). The EMERCOM EMT is WHO classified (EMT-2). For reported capacities of the I-EMT-2s and their medical equipment, see Table 1 and Table 2.

**Table 1.** Characteristics of International Field Hospitals (Equivalent to or Classified as EMT-2) Present in Beirut after the Blast on August 4, 2020

	Iranian RC	Italian	Jordanian	Moroccan	EMERCOM
Total Staff	**27**	**ND**	**117**	**46**	**19**
MD	6		25	21	8
Nurse	16		40	25	10
Specialties	GP, Surgery, Cardiology, Emergency Medicine	ND	Surgery, Pediatrics, Obstetrics, Gynecology^a^	GP, Surgery, Pediatrics, Obstetrics, Gynecology, Plastic Surgery	GP, Surgery, Cardiology, Pediatrics
OPD Services	Yes	ND	ND	8AM-4PM (Except Sunday)	8AM-8PM
Emergency Services (Daytime)	Yes	ND	Yes	Yes	Yes
IPD Beds (Numbers)	Yes (20)	Yes (32)	Yes (48)	Yes (30)	Yes (ND)
ICU Beds (Numbers)	No	Yes (3)	Yes (10)	No	Yes (ND)
OT^b^ (Numbers)	Yes (1)	Yes (1)	Yes (2)	Yes (2)	Yes^c^ (1)
Capacity Deliveries/ C-Section	ND	ND	No	Yes	No
Providing Psychiatric Care	No	ND	No	ND	Yes

Note: Adapted data from the WHO EMT CC and UNFPA Lebanon Emergency Field Hospitals Healthcare Facilities Assessment.

Abbreviations: RC, Red Crescent; EMERCOM, Ministry of Emergency Situations of the Russian Federation; EMT-2, Emergency Medical Team Type 2; GP, general physician; IPD, in-patient department; MD, medical doctor; ND, no data available; OPD, out-patient department; OT, operating theater; ICU, intensive care unit.

a Some data missing from reports, unknown if there were GPs and/or cardiologists.

b With anesthesiologist on duty 24/7 and post-surgical facilities.

c The Russian EMERCOM did not have staffed and equipped post-surgical facilities.

**Table 2.** Medical Equipment Available at the International Field Hospitals (Equivalent to or Classified as EMT-2) Present in Beirut after the Blast on August 4, 2020

	Iranian RC	Italian	Jordanian	Moroccan	EMERCOM
PPE Availability	Yes	ND	Yes	Yes	Yes
Sterilization Unit	No	ND	Yes	Yes	Yes
X-ray/Ultrasound	No	ND	Yes/Yes	Yes/Yes	Yes/Yes
Lab Services	No	ND	Yes	Yes	Yes
PCR/Rapid Tests COVID-19	No	Yes	No	Yes	Yes
Pharmacy and Service Hours	Yes	ND	Yes 8AM – 2PM	Yes	Yes 24 Hours/Day
Transports	Own Ambulance	None	Lebanese Army Ambulance	Ambulance from LRC	Lebanese Army Ambulance

Note: Data adapted from the UNFPA Lebanon Emergency Field Hospitals Healthcare Facilities Assessment.

Abbreviations: RC, Red Crescent; EMERCOM, Ministry of Emergency Situations of the Russian Federation; EMT-2, Emergency Medical Team Type 2; COVID-19, coronavirus disease 2019; LRC, Lebanese Red Crescent; ND, no data available; PCR, polymerase chain reaction; PPE, personal protection equipment.

In addition, four I-EMTs equivalent to Specialized Care Teams were deployed initially to assess health care needs. The teams arrived from Poland (Polish Center for International Aid [PCPM]; Warsaw, Poland), Switzerland (Swiss Agency for Development Cooperation and Humanitarian Aid [SDC]; Bern, Switzerland), the United Kingdom (UK-EMT; Manchester, United Kingdom), and the United States (Samaritan’s Purse; Boone, North Carolina USA). All Specialized Care Teams were civilian: two NGOs and two governmental. The PCPM EMT, the UK-EMT, and the SDC are WHO EMT classified, while Samaritan’s Purse is in the process of classification.

The Qatari army donated two structures that were erected outside two destroyed hospitals with space for approximately 100 hospitals beds as well as medical equipment.

### Activities of I-EMTs

Availability of data on the I-EMT-2 activities varied. The EMERCOM hospital reported around 1,000 consultations without further specification. The Iranian RC field hospital provided mainly out-patient consultations (OPCs); no specific data were made available to the WHO EMT CC. Reportedly, some 300-400 OPCs were done until the structure was donated to local health authorities one month after arrival. The Jordanian field hospital offered OPCs but did not report activities. During a visit by the WHO EMT CC two weeks after the blast, this hospital had not admitted patients nor performed any major explosion-related trauma surgery. Data from the Moroccan military field hospital reported both OPCs and surgeries. The Italian field hospital had 32 beds and intensive care unit (ICU) capacity, but admissions to the facility remained low. A total of 1,100 OPCs and 1,300 polymerase chain reaction (PCR)-tests for COVID-19 were reported, but no data were available on specific activities. During two visits by the WHO EMT CC, daily activities were limited to a few OPCs of mainly non-communicable diseases. All OPC patients were PCR tested for COVID-19 prior to entering the I-EMT-2s. If they tested positive for COVID-19, they were referred to a public hospital. See Table 3 for the data shared by the EMT-2s.

**Table 3.** Total Number of Reported In-Hospital and Out-Patient Activities at the Moroccan Military Field Hospital (Equal to EMT-2) and the EMERCOM Field Hospital (Classified EMT-2)

Minor and Major Surgeries		Examinations		Medical Consultations (Number of Hospitalizations)	
Moroccan	EMERCOM	Moroccan	EMERCOM	Moroccan	EMERCOM
**431** ^**a**^	**388**	**6,433**	**390**	**23,167 (469)**	**700** ^**b**^ **(14)**

Note: Types of injuries – victims of the explosion, traffic accidents, domestic accidents, and incidents of daily life.

Abbreviations: EMERCOM, Ministry of Emergency Situations of the Russian Federation; EMT-2, Emergency Medical Team Type 2.

a Including four deliveries.

b Of which, 69 were urgent psychological care.

The I-EMT Specialized Care Teams deployed to support local agencies and hospitals and did not report specific activities. Based on identified needs, three I-EMT Specialized Care Teams were re-tasked by the WHO EMT CC to provide technical support for COVID-19 care at six public hospitals.

The two prefabricated structures set up by the Qatari army did not have running water nor toilets. When visited by the WHO EMT CC a month after the blast, the structures had not been used for clinical practice.

### Timing of I-EMTs

**Figure 1.**
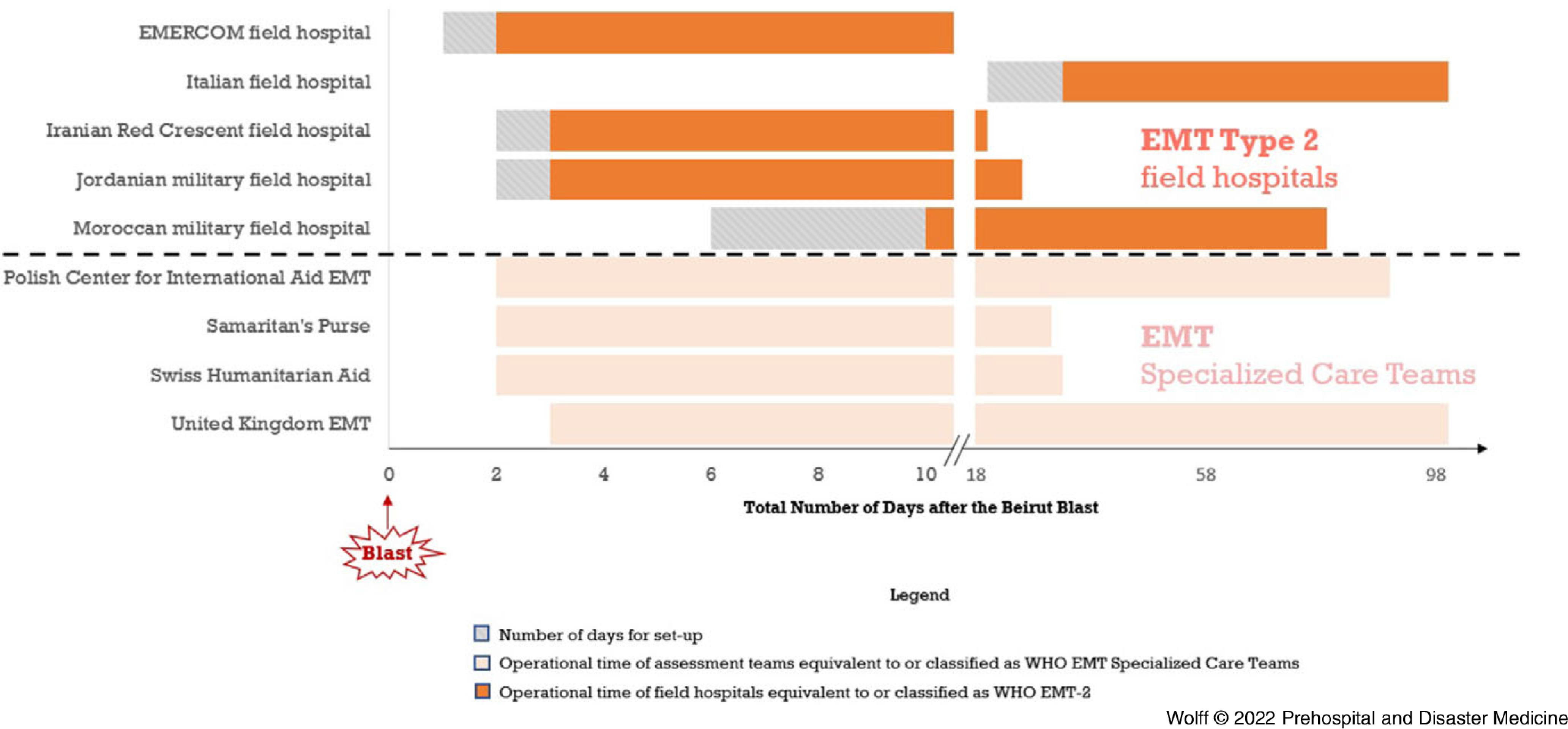
Timing of I-EMTs. Abbreviations: EMERCOM, Ministry of Emergency Situations of the Russian Federation; EMT, Emergency Medical Team; I-EMT, International EMT; WHO, World Health Organization.

The timing of the I-EMTs varied (Figure 1). The majority arrived within days of the explosion. The EMERCOM hospital had the shortest stay: 10 days. The Italian and Moroccan hospitals stayed the longest of all I-EMT-2s: for two and two-and-a-half months, respectively (IQR 98 days for all the I-EMTs present in Beirut).

The SDC and the team from Samaritan’s Purse stayed for approximately one month. The PCPM stayed until the beginning of November 2020. The UK-EMT supported COVID-19 care at four public hospitals in Lebanon until March 2021.

The structures that the Qatari army donated were erected two weeks after the blast.

## Discussion

A majority of I-EMTs were focused on providing trauma care, though these needs had already been met by the national health system. The well-developed (albeit financially struggling) Lebanese health care has significant experience in treating explosion- and conflict-injured. The explosion mainly generated minor injuries that could be managed ambulatory; only around 12% of the injured needed hospitalization.^5^ Three hospitals were destroyed in Beirut (corresponding to a loss of 5% of hospital beds in the country), while remaining hospitals were intact.^3^ Primary health care was needed as almost 40% of primary care facilities in the affected area were damaged in the blast.^18^ Previous studies and guidelines have emphasized the need for multi-disciplinary EMT capacities in order to also cover the normal burden of disease.^13,15^ However, most of the I-EMT-2s in Beirut did not expose such capacity. At the time of the blast, few COVID-19 cases were hospitalized in Lebanon, something that changed in the following months.^19^ None of the I-EMT-2s accepted nor could adapt their services to include care for COVID-19 patients, despite requests from the WHO EMT CC.

Most of the deployed I-EMT-2s were military. Military field hospitals have resources to rapidly deploy to disasters where national health care is strained. In Beirut, the Moroccan military field hospital provided health care corresponding to the normal burden of disease in Lebanon. This hospital was also located close to the most affected area and rendered positive appreciation from the local community. The Jordanian field hospital was mainly trauma oriented and located within a military zone, which hindered easy access for the general population. The Italian field hospital arrived to provide trauma care, but the hospital remained largely empty, its resources were not optimally used. This suggests that military field hospital deployment must be agile and adapted to medical needs and available capacities.

A significant lack of reporting made it difficult to perform an in-depth analysis of the I-EMT-2s activities in relation to medical needs. A minimum data set for EMT reporting is available^20^ and the EMT initiative emphasizes transparency. Still, non-reporting is known in previous studies.^6,7,9–11,17^ None of the I-EMT-2s in Beirut reported their activities to the WHO EMT CC. After several attempts by the EMT CC and months after the blast, two of the EMT-2s provided basic data on their activities. Data could not be verified nor validated, making interpretation difficult. The activities reported remained low compared to reported capacities. The EMT Specialized Care Teams adhered to EMT standards and attended weekly EMT CC meetings where they reported their non-clinical activities. For future EMT deployments, it is essential to ensure systematic reporting mechanisms and that this remains a EMT core standard.

## Limitations

As in previous studies, the I-EMT data available were limited, which made an in-depth analysis of the I-EMT activities impossible. Moreover, the quality of data was not possible to validate. Data were only presented that were made available to the EMT CC. The lack of self-reported data risks introducing selection bias. To balance this, WHO visited and met with staff as well as observed activities at all five I-EMT-2 facilities. The deployed international field hospitals are invited to share ideas on improving future I-EMT data sharing and responses.

## Conclusion

The international health care response to the Beirut blast was significant and mainly focused on trauma care, while the need for such care was limited when the five field hospitals (EMT-2s) arrived. A majority of the EMT-2s were military. The four other EMTs were adapted to support the existing health system; three of them were re-purposed to support COVID-19 care at public hospitals. Availability of data remains an obstacle to assess EMT activities and suggests improvements.
